# Uptake of and Motivational Responses to Mental Health-Promoting Practices: Comparing Relaxation and Mindfulness Interventions

**DOI:** 10.3389/fpsyg.2022.869438

**Published:** 2022-07-14

**Authors:** Marguerite M. Beattie, Nelli E. Hankonen, Hanna M. Konttinen, Salla-Maarit Volanen

**Affiliations:** ^1^Social Psychology, Faculty of Social Sciences, University of Helsinki, Helsinki, Finland; ^2^Social Psychology, Faculty of Social Sciences, University of Tampere, Tampere, Finland; ^3^Sociology, Faculty of Social Sciences, University of Helsinki, Helsinki, Finland; ^4^Department of Public Health, Faculty of Medicine, University of Helsinki, Helsinki, Finland; ^5^Folkhälsan Research Center, Helsinki, Finland

**Keywords:** reasoned action approach, behavior change, mental health, adolescents, intervention acceptability, mindfulness practice, relaxation practice

## Abstract

**Background:**

Comparative analyses of alternative interventions within the same trial enable acceptability and fidelity of each to be investigated more critically. In addition, whereas so far studies have focused on efficacy evaluations, more understanding is needed on motivational factors influencing the uptake of mental health-promoting practices rather than solely their effects.

**Purpose:**

This study investigates whether the motivational responses to a mindfulness intervention are different from a relaxation intervention. We compare social cognitions outlined by the reasoned action approach and their roles in practice uptake, self-reported reasons for non-practice, and experienced benefits.

**Methods:**

In a cluster-randomized trial (ISRCTN18642659; *N* = 3134), 12–15-year-old participants were given a 9-week intervention and followed up to 52 weeks. Main statistical analyses included *t*-tests, mixed ANOVAs, path models, and chi-square tests.

**Results:**

Social cognitions in the mindfulness arm were slightly more positive immediately post-intervention, but recipients mostly responded similarly to the two interventions in the longer term. While attitudes, norms, intention, and self-efficacy were relatively high post-intervention, most of them slightly decreased by 26 weeks. Main reasons for non-practice in both arms included not finding the exercises helpful, no felt need, boringness of exercises and forgetting. The most common benefits experienced by practicing respondents were stress management and concentration ability. Better sleep was a more frequently reported benefit in the relaxation arm, but no other major differences emerged.

**Conclusion:**

This study offers an example of comparing motivational responses to experimental and active control arm interventions, a potentially helpful approach in improving intervention adherence.

## Introduction

Interventions have attempted to promote mental health *via* several different practices, such as engaging in relaxation practices or practicing mindfulness. While intervention studies have examined effectiveness on mental health outcomes, evaluations rarely investigate social cognitive responses (e.g., attitudes, norms, and intention to practice) and the uptake of practice after the intervention, or their changes in the long-term. Moreover, evaluations of mental health-promoting interventions often fall short of making sense out of participant engagement due to an absence of a meaningful comparison arm. Usually control arms in such trials are passive, i.e., are not subjected to any other alternative, active treatment. Therefore, the levels of the resulting attitudes, motives, strength of motivation, etc., have no reasonable comparison point, a “benchmark,” which would aid in making better sense of the results.

In order for health behavior interventions to have their intended effects, the intervention has to be well received by the participants: They need to be motivated to enact the skill taught in interventions later in their daily lives (Bellg et al., 2004). If this chain of implicit assumptions in RCTs does not occur, conclusions drawn from the trial may not be valid. Unfortunately, there is a dearth of research into receipt and enactment of health behavior change interventions (Rixon et al., 2016; Walton et al., 2017; Hankonen, 2020). This study has a unique opportunity to compare and contrast social cognitive responses to mental health promoting interventions and asks: Are the motivation base and cognitive response to mindfulness practices essentially different from relaxation practices, in a school-based intervention? Do motivational factors predict similarly both types of practice?

We study several motivational, social cognitive factors that could potentially explain practice behavior, based on the reasoned action approach (RAA). Motivation to practice, or motivational responses to interventions can be studied from the perspectives of (1) social cognitive determinants of motivation, e.g., the RAA constructs, and (2) perceived reasons for non-practice and benefits experienced. The RAA posits that attitudes, perceived norms, and perceived behavioral control/self-efficacy regarding the behavior predict intention to perform the behavior, which predicts performance of the behavior. In adolescent populations, a particularly strong link has been found between descriptive norms and behavior (McEachan et al., 2016). Attitudes toward the behavior, perceived norms, and perceived behavioral control have been found to be associated with intention to meditate (Lederer and Middlestadt, 2014; Beattie et al., 2019, 2020; Erbe et al., 2019). However, to date, there is still a dearth of empirical evidence, however, on what factors predict mindfulness and relaxation practice at home for adolescents in spite of low adherence (Zenner et al., 2014; Felver et al., 2016). Due to the lack of previous research, our research is exploratory, and we did not form hypotheses about what the differences would be. We may conjecture that there could be differences, e.g., mindfulness practice may be seen as more foreign, hippie, or religious or may be experienced as more difficult, even frustrating.

Secondly, motivation to practice can also be studied from the perspective of reasons for not practicing (e.g., barriers), and perceived benefits. As for reasons for not practicing, time constraints are one of the most frequently found reasons in previous research (Gryffin et al., 2014; Laurie and Blandford, 2016; Kerr et al., 2019; Toivonen et al., 2020). As for experienced benefits post-intervention, improvements in relationships and stress management have been reported in adult populations (Parra et al., 2019; Erbe et al., 2020). However, there are more that have not been studied, potentially specific to an adolescent student population (e.g., concentration when involved in school). Reasons for not practicing and benefits are conceptually similar to RAA constructs, but unlike the RAA constructs, they are explicitly stated as reasons for not practicing and benefits experienced from having practiced. In other words, participants themselves report them as preventing practice or resulting from practice (respectively); by contrast, RAA constructs are tested as being related to practice statistically.

The authors’ previous work has found the RAA to be applicable to the prediction of mindfulness uptake in the same Healthy Learning Mind trial (Beattie et al., 2020). Here we extend the analyses to the relaxation arm and compare predictors of practice uptake in the same trial. This will help make sense out of the immediate evaluative, cognitive response of mental health-promoting interventions, which can be a sign of intervention acceptability and successful receipt (Rixon et al., 2016). In order to understand the specific, explicit barriers and benefits the participants perceive, the present study examines these in addition to the RAA.

We investigate, in a longitudinal follow-up of two active trial arms:

*Social cognitive and behavioral responses and changes over time*

Are there differences in social cognitions (outcome expectations, perceived norms, self-efficacy, and intentions) and practice between the arms and across time?

Does the RAA predict intention and behavior for both relaxation and mindfulness practice similarly?

*Self-reported motives and gained benefits*

What reasons do youth self-report for non-practice, what benefits do the youth report from practicing, and do the arms differ?

## Method

### Participants and Design

The cluster-randomized trial Healthy Learning Mind (ISRCTN18642659) evaluated comparative effectiveness of mindfulness, relaxation, and non-treatment arms on psychological outcomes in 56 schools and the primary evaluation has been published (Volanen et al., 2020). The current study analyzes data from the participants in the two active arms: mindfulness (*n* = 1,646; *k* = 94) and relaxation (*n* = 1,488; *k* = 85). Surveys were administered at baseline (0 weeks), 10, 26, and 52 weeks. For further details, see the study protocol (Volanen et al., 2016).

### Interventions

Both interventions took place in weekly 45-min sessions in school for 9 weeks. In the experimental arm, participants were taught mindfulness techniques [based on the. b program (Kuyken et al., 2013)], e.g., breath counting, observing thoughts and bodily sensations, and awareness in everyday tasks. In the active control arm (i.e., relaxation), exercises included, e.g., progressive muscle relaxation, a breathing exercise, visualization, choose your emotion for the rest of the day and a short break for regaining energy (Volanen et al., 2016).

### Measures

The measures are described in Supplementary Table 1. The social cognitive variables were constructed with guidance from Francis et al. (2004). Injunctive norms were measured by two items about friends’ and parents’ approval of practice, and descriptive norms by one item about friends’ practice. Intention was measured with a single item about intention to practice in the following months. As for the practice measures, the follow-up surveys asked participants to report their use of short and long exercises during the past month and the past half year resulting in four items for each arm. The practice measures were similar for the two arms; the only differences were that the exercises were labeled as breathing or relaxation exercises and examples were given for the mindfulness arm. The surveys also provided six choices of reasons the participants could check for not practicing, and eight choices of benefits on a Likert scale.

### Statistical Analyses

For the continuous variables, mean differences between the two arms were assessed using independent *t*-tests, and between time points with paired *t*-tests. Mean differences by group and time were assessed with Mixed ANOVAs. Holm–Bonferroni sequential correction was applied to correct for multiple comparisons. Differences between the trial arms in reasons for not practicing were analyzed using chi-square tests, Bayes factors (multinomial models with fixed rows since the number in each arm was fixed), and odds ratios.

The RAA path model was tested using a multi-group path analysis, adjusted for grade level. The measure of practice chosen for this analysis was practice of short exercises during the past half year because it had the lowest skewness and kurtosis scores (see Supplementary Table 2). Standard errors and confidence intervals were adjusted for clustering at the class level. A chi-square difference test was used to evaluate whether the RAA model paths varied across trial arms. More specifically, the chi-square statistic of the constrained model (the regression paths were forced to be similar between groups) was compared with that of the unconstrained model (the paths were allowed to vary freely) using the Satorra–Bentler scaled chi-square difference test. Full Information Maximum Likelihood estimator with robust standard errors was applied to handle missing data (the lowest covariance coverage between variables was 44% for mindfulness participants and 50% for relaxation participants) and to take into account deviations from normality and non-independence of observations.

Analyses were conducted using IBM SPSS Statistics 24/25 with the following exceptions: multi-group path analyses were performed with Mplus Version 7 and effect sizes not given by SPSS were calculated with online calculators (Wiseheart, 2018; Stangroom, 2021). We used 0.01 rather than the common 0.05 cut-off due to a relatively large sample size. To evaluate effect sizes, we used the references recommended by Cohen (1988). Skewness (>2) and kurtosis (>7) cutoffs were based on Finney and DiStefano (2006) recommendations. The model fit was evaluated with several types of fit indexes including the chi-square statistic, Tucker-Lewis Index (TLI), Comparative Fit Index (CFI), and Root Mean Square Error of Approximation (RMSEA). TLI and CFI values ≥ 0.95 and RMSEA values ≤ 0.06 were defined to indicate a good fit (Hu and Bentler, 1999).

## Results

### Social Cognitive and Behavioral Responses and Changes Over Time

There were significant albeit small differences between the arms at 10 weeks with the mindfulness arm showing a systematically more positively inclined social cognitive response: higher positive outcome expectations (*d* = 0.16), lower negative outcome expectations (*d* = –0.14), higher self-efficacy (*d* = 0.14), higher injunctive norms (*d* = 0.20), higher descriptive norms (*d* = 0.20), and higher intention (*d* = 0.25) compared to the relaxation arm. At 26 weeks, there were no statistically significant differences between the arms. From 10 to 26 weeks, neither arm had significant changes in positive or negative outcome expectations or self-efficacy (*d* = 0.00–0.11). However, both arms did have significant decreases in injunctive and descriptive norms and intention (*d* = [–0.10] – [–0.54]). The only interaction effects between time and group were found in descriptive norms (*p* < 0.001) and intention (*p* = 0.001) with those factors decreasing more in the mindfulness arm. However, the interaction effects were quite small (η*_*p*_*^2^ = 0.007 for both descriptive norms and intention). See Table 1 for more details.

**TABLE 1:** Differences in social cognitive variables.

		Mindfulness								Relaxation											
			
								Paired *t*-test								Paired *t*-test		Independent *t*-Test		Mixed ANOVA	
																	
Social cognitive variable	Time	Mean	SD	MIN	MAX	Skewness	Kurtosis	*p*	*d* ^a^	Mean	SD	MIN	MAX	Skewness	Kurtosis	*p*	*d^a^*	*p*	*d^a^*	*P*-value	η_*p*_^2^
Positive outcome expectancies	10 Weeks	3.73	0.86	1	5	–0.756	0.910			3.59	0.94	1	5	–0.633	0.495			0.001*	0.16		
								0.958	<0.01							0.927	<0.01			0.672	0.000
	26 Weeks	3.75	0.89	1	5	–0.759	0.848			3.62	0.93	1	5	–0.612	0.394			0.006	0.14		
Negative outcome expectancies	10 Weeks	2.39	1.06	1	5	0.356	–0.549			2.54	1.05	1	5	0.317	–0.321			0.002*	–0.14		
								0.006*	0.11							0.034	0.08			0.917	0.000
	26 Weeks	2.50	1.01	1	5	0.280	–0.382			2.64	1.02	1	5	0.192	–0.387			0.006	–0.14		
Self-efficacy	10 Weeks	2.96	0.61	1	4	–0.387	0.606			2.87	0.67	1	4	–0.422	0.401			0.003*	0.14		
								0.334	0.03							0.121	0.05			0.625	0.000
	26 Weeks	2.98	0.60	1	4	–0.506	1.006			2.92	0.64	1	4	–0.568	0.894			0.019	0.10		
Injunctive norms	10 Weeks	3.68	1.04	1	5	–0.542	0.034			3.47	1.10	1	5	–0.380	–0.164			<0.001**	0.20		
								<0.001**	–0.17							0.005*	–0.10			0.202	0.001
	26 Weeks	3.51	1.78	1	5	–0.443	–0.384			3.37	1.23	1	5	–0.411	–0.537			0.016	0.09		
Descriptive norms	10 Weeks	2.46	1.10	1	5	0.047	–0.899			2.24	1.14	1	5	0.422	–0.732			<0.001**	0.20		
								<0.001**	–0.45							<0.001**	–.046			<0.001**	0.007
	26 Weeks	1.99	1.09	1	5	0.767	–0.268			1.93	1.07	1	5	0.776	–0.399			0.254	0.06		
Intention	10 Weeks	3.55	1.88	1	7	–0.007	–1.19			3.09	1.86	1	7	0.361	–1.10			<0.001**	0.25		
								<0.001**	–0.54							<0.001**	0.39			0.001*	0.007
	26 Weeks	2.64	1.74	1	7	0.707	–0.613			2.46	1.69	1	7	0.774	–0.629			0.018	0.10		

*^a^Cohen’s d from http://www.socscistatistics.com/effectsize/Default3.aspx and http://www.cognitiveflexibility.org/effectsize/effectsizecalculator.php.*

*P-value = 0.01, **P-value = 0.001 after Holm-Bonferroni sequential correction.

Whereas immediately after the intervention, 33.7% of respondents in the mindfulness arm and 23.6% in the relaxation arm agreed fully/partially in the intention question, only 14.5 and 13.2% (respectively) did at the 26 weeks follow-up. This demonstrates the drop in the proportion of motivated individuals to engage in the mental health-promoting practices after a period without any boosters.

As for behavioral practice variables changes over time, from 26 to 52 weeks, only *short mindfulness exercises* in the past half year decreased slightly (*r* = –0.16). No changes were detected in other behaviors (i.e., all *relaxation* exercises, *long mindfulness exercises during the past half year* and *4 weeks*, and s*hort mindfulness exercises during the past 4 weeks*; *r* = [–0.07] – [0.03]). Comparing the mindfulness to relaxation arms, they only statistically significantly differed at 26 weeks in long and short exercises during the past half year, with the relaxation arm reporting less practice. See Supplementary Table 2 for more details.

### Path Models

**FIGURE 1:**
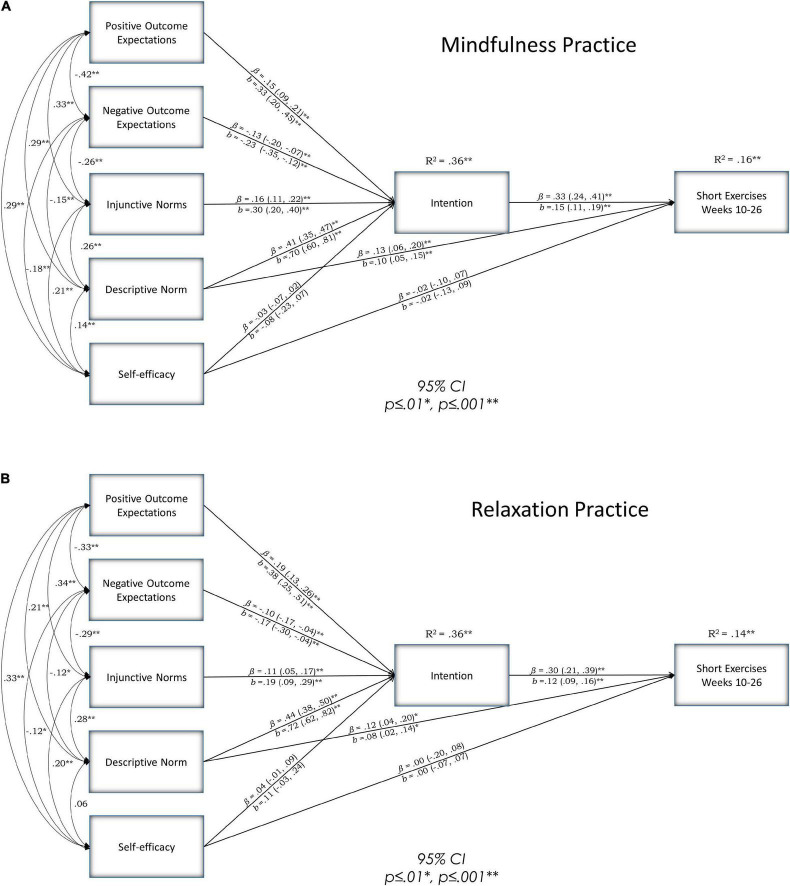
Multi-group path models: **(A)** Mindfulness and panel **(B)** relaxation. Results 95% CI, *p* ≤ 0.01*, *p* ≤ 0.001^**^. The model was adjusted for grade level, i.e., specified to predict intention and practice. Multi-group modeling was used to produce separate parameter estimates for the relaxation and mindfulness arms. All variables except for the practice variables come from the 10-week survey. The practice variables come from the 26-week survey.

The RAA Path Model had a good fit for the data χ***^2^***(6, *N* = 3134) = 13.055, *p* = 0.042, TLI = 0.969, CFI = 0.993, and RMSEA = 0.967. The associations within the model did not differ across the arms (Δχ^2^ = 6.97, *p* = 0.539). Outcome expectations and injunctive and descriptive norms were predictive of intention but self-efficacy was not. Descriptive norms were the strongest predictor of intention. Similarly, intention and descriptive norms were predictive of practice but perceived behavioral control was not. See Figure 1 for direct effects and Supplementary Table 3 for indirect effects.

### Self-Reported Motives and Gained Benefits

As for the self-reported reasons for not practicing, “not finding the exercises helpful” was the most frequently chosen reason in both arms and both timepoints, with about half of the respondents reporting this. There were no differences between the arms. See Table 2 for more details. The most common experienced benefits by practicing respondents were “stress management” and “concentration ability.” For both arms at 26 and 52 weeks, average levels of perceived benefits did not reach 3 on a scale from 1 to 5. There were no statistically significant changes over time for either arm in any of the benefits (all *d* < ∓0.1). Between the arms, only the benefit of better sleep at 52 weeks was significantly different with the relaxation arm experiencing it more (*d* = –0.24; others *d* < ∓0.19). There were no interaction effects between time and group (all *p* ≥ 0.091 and all η*_*p*_*^2^ < 0.005). See Table 3 for more details.

**TABLE 2:** Differences in reasons for not practicing.

Reasons for not practicing		Percentages (row)		Pearson’s chi-square			Bayes factor_01_
			
		*Mindfulness*	*Relaxation*	*χ^2^*	df	*p*	
I didn’t find them helpful	26 Weeks	41.7%	46.9%	5.419	1	0.022	1.2
	52 Weeks	49.3%	47.5%	0.392	1	0.562	11.3
The exercises were too difficult	26 Weeks	3.5%	4.3%	0.915	1	0.360	29.3
	52 Weeks	4.6%	5.6%	0.705	1	0.432	22.1
I have forgotten to do the exercises	26 Weeks	27.6%	24.4%	2.597	1	0.115	5.6
	52 Weeks	26.9%	23.9%	1.442	1	0.232	7.7
I have been too busy to do the exercises	26 Weeks	19.6%	21.9%	1.721	1	0.206	9.3
	52 Weeks	20.7%	25.6%	4.026	1	0.047	2.2
I think the exercises are boring	26 Weeks	22.0%	25.8%	3.882	1	0.053	3.0
	52 Weeks	26.6%	26.1%	0.043	1	0.844	15.3
I have not needed the exercises	26 Weeks	26.8%	29.3%	1.532	1	0.233	9.3
	52 Weeks	26.3%	30.7%	2.907	1	0.095	3.6

**TABLE 3:** Differences in benefits experienced*^ab^*.

Benefits experienced	Time	Mindfulness						Relaxation									
			
						Paired T-test						Paired T-test		Independent T-Test		Mixed ANOVA	
													
		Mean	SD	Skewness	Kurtosis	*p*	*d^c^*	Mean	SD	Skewness	Kurtosis	*p*	*d^c^*	*p*	*d^c^*	*p*	η_*p*_^2^
Concentrate better in class	26 Weeks	2.17	1.22	*0.66*	*−0.62*	0.132	−0.085	2.17	1.21	*0.58*	*−0.77*	0.456	−0.038	*0.998*	0.00	0.111	0.004
	52 Weeks	1.95	1.21	*0.94*	*−0.26*			2.15	1.26	*0.69*	*−0.63*			*0.014*	−0.16		
Concentrate better on hobbies	26 Weeks	2.23	1.28	*0.61*	*−0.80*	0.313	−0.053	2.19	1.28	*0.62*	*−0.81*	0.475	−0.036	*0.501*	0.03	0.226	0.002
	52 Weeks	1.99	1.24	*0.90*	*−0.39*			2.18	1.28	*0.65*	*−0.76*			*0.031*	−0.15		
Manage stress better	26 Weeks	2.26	1.26	*0.56*	*−0.85*	1.000	< −0.001	2.26	1.26	*0.51*	*−0.92*	0.406	−0.058	*0.955*	0.00	0.561	0.001
	52 Weeks	2.07	1.26	*0.79*	*−0.57*			2.27	1.32	*0.55*	*−0.95*			*0.039*	−0.15		
Cope better with difficult emotions, e.g., fear, anger, aggression, and anxiety	26 Weeks	2.16	1.23	*0.68*	*−0.61*	0.105	−0.094	2.19	1.27	*0.65*	*−0.74*	0.719	−0.016	*0.597*	−0.02	0.377	0.001
	52 Weeks	1.92	1.18	*0.97*	*−0.17*			2.16	1.25	*0.63*	*−0.75*			*0.004*	−0.19		
Sleep better	26 Weeks	2.18	1.29	*0.68*	*−0.76*	0.260	−0.063	2.24	1.29	*0.57*	*−0.88*	0.207	0.070	*0.382*	−0.05	0.091	0.005
	52 Weeks	1.95	1.24	*0.95*	*−0.37*			2.26	1.29	*0.53*	*−0.94*			0.000**	−0.24		
Get better grades on exams	26 Weeks	2.05	1.22	*0.82*	*−0.46*	0.367	−0.053	2.09	1.21	*0.71*	*−0.59*	0.790	−0.015	*0.508*	−0.03	0.658	<0.001
	52 Weeks	1.85	1.17	*1.08*	*0.02*			2.09	1.25	*0.78*	*−0.51*			*0.004*	−0.19		
Get along better with my friends	26 Weeks	2.05	1.25	*0.86*	*−0.42*	0.470	−0.039	2.10	1.27	*0.77*	*−0.61*	659	−0.023	*0.378*	−0.04	0.854	<0.001
	52 Weeks	1.86	1.19	*1.08*	*−0.02*			2.09	1.27	*0.78*	*−0.58*			*0.007*	−0.18		
Get along better with my family members	26 Weeks	2.04	1.24	*0.88*	*−0.37*	0.351	−0.056	2.11	1.26	*0.76*	*−0.59*	0.751	−0.023	*0.332*	−0.06	0.675	<0.001
	52 Weeks	1.86	1.19	*1.08*	*−0.03*			2.09	1.26	*0.77*	*−0.55*			*006*	−0.18		

*^a^Means and standard deviations are based on all available cases rather than the cases available for each test. Therefore, the means do not correspond to each test. For example, the cases available for the change in managing stress for the mindfulness arm (n = 187) were a lot fewer than the total available cases (n_26wk_ = 607; n_52wk_ = 364). The means are the same at both time points for the cases available for the test, whereas the means are different for the total cases available.*

*^b^Min-Max:1–5 for all variables.*

*^c^Cohen’s d from http://www.socscistatistics.com/effectsize/Default3.aspx and http://www.cognitiveflexibility.org/effectsize/effectsizecalculator.php.*

*P-value ≤0.01, **P-value ≤0.001 after Holm–Bonferroni Sequential Correction.

## Discussion

This study set out to investigate social cognitive, motivational responses to two interventions teaching mental health-promoting practices to young adolescents. These are indicators of both the receipt (or even acceptability) and the future continuation of the practice behavior. We found that, on average, participants of both relaxation and mindfulness interventions reported moderately high positive outcome expectations for engaging in the taught practices, moderate negative outcome expectations, moderately high injunctive norms (friends’ and parents’ approval), moderately low descriptive norms (friends’ behavior), and moderate self-efficacy. Immediately post-intervention, social cognition regarding mindfulness was more positive in the mindfulness arm compared to the relaxation arm. However, these differences seemed to disappear; the decline in these variables was stronger for the mindfulness arm (except the descriptive norm), rendering the mean levels similar at 26 weeks. These results then do not show differences in injunctive norms as may have been expected if mindfulness were perceived as more foreign, hippie, or religious, or in self-efficacy if mindfulness exercises were perceived as more difficult.

It should be noted, that although there were slight or major decreases in most social cognitive variables, the means of positive outcome expectancies and self-efficacy remained the same at follow-up. The driver in the major drop for intention, thus, may mostly reside in negative outcome expectations and normative beliefs. Descriptive norms showed the greatest drop, possibly due to the active intervention sessions at school having ended. It should also be noted that whereas “short mindfulness exercises” decreased more over time than did other forms of behavior, this may be due to a floor effect for the other behaviors rather than that the participants would keep up these other forms of exercises more than the short mindfulness ones: Short mindfulness exercises were higher to begin with. Also, the size of decrease was not very large, rendering practical significance of the decrease questionable (from 1.55 to 1.42).

This is the first study to compare social cognitive responses related to the mental health-promoting exercises (relaxation vs. mindfulness), and how they predict later behavior, to our knowledge. Evaluation of engagement with (fidelity of receipt and enactment) and acceptability of mental health-promoting programs is important across different phases of behavioral trials: (1) In pilot and feasibility trials, investigating responses gives important information for intervention optimization (feasibility and optimization). (2) Process evaluations in general can help interpret main outcomes of a trial (definitive trial phase). (3) Finally, as universal mental health-promoting programs are being increasingly rolled out nation-wide, we need to know what target participants’ (likely) attitudes toward these programs are (implementation phase; Skivington et al., 2021). This study in particular provides information for intervention optimization.

Limitations of this study include self-report measures and partly suboptimal operationalizations of the RAA constructs [see section “Discussion” in Beattie et al. (2019)]. Strengths include a multifaceted investigation of various indicators of receipt and the design, which enabled a comparison of two active intervention arms. Secondly, the sample size allowed for reliable comparative investigations, rarely available thus far. Thirdly, it should be noted that these results about reasons for not practicing should be taken with caution as missing responses and not choosing the responses deliberately cannot be distinguished.

For mental health-promoting programs, it may be useful to consider the communication within the programs in terms of likely effects on recipients’ self-efficacy, attitudes toward the behavior, and perceived norms. Such factors may influence enrollment into mindfulness programs as well as uptake and sustained use of mindfulness practices. In this intervention, there was room for all social cognitions to be improved by at least a point on the scale.

As for implications for future research, motivation and self-regulation theories may present an interesting addition to studying mental health promoting behaviors: they may pave the way for improved effectiveness of interventions through effectively intervening on predictors of practice behaviors (Hagger et al., 2020). These kinds of findings can inform the design of interventions to improve fidelity (Beattie et al., 2019). Improved attention to fidelity (receipt and enactment) of behavior change intervention trials helps acknowledge and tackle possible threats to trial validity (Toomey et al., 2020).

This study shed light on similarities and differences in reception and acceptability of two mental health-promoting interventions, through the lens of social cognitions and perceived benefits and barriers. Essentially, the results imply that both mindfulness and relaxation exercises are similarly acceptable, and have similar perceived benefits and the barriers for practice.

## Data Availability Statement

The raw data supporting the conclusions of this article will be made available by the authors, without undue reservation.

## Ethics Statement

The studies involving human participants were reviewed and approved by Humanities and Social and Behavioral Sciences Ethical Review Board of the University of Helsinki (Statement 1/2014). Written informed consent to participate in this study was provided by the participants or their legal guardian/next of kin.

## Author Contributions

MB: conceptualization, formal analysis, writing – original draft and review and editing, funding acquisition, and methodology. NH: conceptualization, writing – original draft and review and editing, supervision, funding acquisition, and methodology. HK: methodology, formal analysis, writing – review and editing, supervision, and conceptualization. S-MV: investigation, resources, writing – review and editing, supervision, and funding acquisition. All authors contributed to the article and approved the submitted version.

## Conflict of Interest

The authors declare that the research was conducted in the absence of any commercial or financial relationships that could be construed as a potential conflict of interest.

## Publisher’s Note

All claims expressed in this article are solely those of the authors and do not necessarily represent those of their affiliated organizations, or those of the publisher, the editors and the reviewers. Any product that may be evaluated in this article, or claim that may be made by its manufacturer, is not guaranteed or endorsed by the publisher.
