# Supplementary material for: Uptake of and Motivational Responses to Mental Health-Promoting Practices: Comparing Relaxation and Mindfulness Interventions
Source: Front Psychol. 2022 Jul 14;13:869438. doi: 10.3389/fpsyg.2022.869438 (PMC9335365; doi:10.3389/fpsyg.2022.869438)
Supplement: Supplementary file 1 [file Data_Sheet_1.docx]

Electronic Supplementary Materials

**Supplementary Table 1.** *Measures*

| **Measures** | **Time Point**  **(week)** | ***n_mindfulness_***  ***n_relaxation_*** | **# of Items** | **Examples of items** | **Type of Scale** |
| --- | --- | --- | --- | --- | --- |
| Positive Outcome Expectancies  (α = .91 - .92) | 10 | 944  921 | 4 | The ability to relax and calm my mind when I’m stressed, nervous or anxious…  … Can help me feel better.  …Can help me perform better (e.g., in sports). | 5-point Likert Scale from  “Totally disagree” to “Totally agree” (Sixth point “I don’t know” was omitted.) |
| Negative Outcome Expectancies  (ρ = .84 - .85) | 10 | 977  926 | 2 | The ability to relax and calm my mind when I’m stressed, nervous or anxious…  …Does not help me in any way. | 5-point Likert Scale from  “Totally disagree” to “Totally agree” |
| Perceived Behavioral Control  (α = .85) | 10 | 1132  1053 | 4 | I think I can calm my mind, even when…  …I have to perform in sports, music, etc., outside school  …I have quarrelled with someone/some people who are close to me | 4-point Likert Scale from  “I am certain I cannot” to “I am certain I can” |
| Injunctive Norms  (ρ = .87-.91) | 10 | 1111  1021 | 2 | My friends think it is OK that I do the home exercises.  My parents think it is OK that I do the home exercises. | 5-point Likert Scale from  “Totally disagree” to “Totally agree” |
| Descriptive Norm | 10 | 1127  1045 | 1 | My friends do some of the exercises we learned. | 5-point Likert Scale from  “Totally disagree” to “Totally agree” |
| Intention | 10 | 1131  1044 | 1 | During the next months, I will use the exercises I have learned to relax and calm my mind. | 7-point Likert Scale from  “Totally disagree” to “Totally agree” |

| **Measures** | **Time Point**  **(week)** | ***n*** | **# of Items** | **Examples of items** | **Type of Scale** |
| --- | --- | --- | --- | --- | --- |
| Mindfulness  Practice  Weeks  10-26  &  27-52 | 26  &  52 | 938  553 | 2 | During the past half year (the time after the Well-being Learning Programme), I did the following exercises at home… Short breathing exercises that lasted under 10 minutes (E.g., FOFBOC, .b, breathing 7/11) | 5-point scale ranging from “Not once” to “Many times a day” |
| Mindfulness  Practice  Weeks  23-26  &  49-52 |  | 938  552 |  | During the past one month (four weeks), I did the following exercises at home…Long breathing exercises that lasted over 10 minutes (Beditation, Feeling my feet on the floor) |  |
| Relaxation  Practice  Weeks  10-26  &  27-52 | 26  &  52 | 980  566 | 2 | During the past half year (the time after the Well-being Learning Programme), I did the following exercises at home… Short relaxation exercises that lasted under 10 minutes | 5-point scale ranging from “Not once” to “Many times a day” |
| Relaxation  Practice  Weeks  23-26  &  49-52 |  | 981  562 |  | During the past one month (four weeks), I did the following exercises at home… Long relaxation exercises that lasted over 10 minutes |  |

| **Measures** | **Time Point**  **(week)** | ***n_mindfulness_***  ***n_relaxation_*** | **# of Items** | **Examples of items** | **Type of Scale** |
| --- | --- | --- | --- | --- | --- |
| Reasons for Not Practicing | 26 & 52 | 1646  1488 | 7 | I have not done mindfulness exercises because  …  I have not needed the exercises | Categorical |
| Benefits Experienced | 26 & 52 | 607-792^a^  364-428^b^  636-762^c^  348-441^d^ | 9 | Because of the doing the [mindfulness or relaxation] exercises I…Concentrate better while in class. | 5-point Likert Scale from  “Not true at all” to “Very true” |

Note. The number for “Benefits Experienced” is given in ranges for each item, because the separate items are used in the analyses. Other measures are averages.

^a^Mindfulness arm at 26 weeks. ^b^Mindfulness arm at 52 weeks. ^c^Relaxation arm at 26 weeks. ^d^Relaxation arm at 52 weeks.

**Supplementary Table 2.** *Differences in Practice Variables^a^*

| Practice Variables | Time | Mindfulness | | | | | |  | Relaxation | | | | | |  | Mann-Whitney U Test | |
| --- | --- | --- | --- | --- | --- | --- | --- | --- | --- | --- | --- | --- | --- | --- | --- | --- | --- |
|  |  |  | | | | **Wilcoxon**  **Signed-Rank**  **Test** | |  |  | | | | **Wilcoxon**  **Signed-Rank**  **Test** | |  |  |  |
|  |  | Mean | SD | Skewness | Kurtosis | *p* | *r* |  | Mean | SD | Skewness | Kurtosis | *p* | *r* |  | *p* | *r* |
| Short Exericses Past Half Year | 26 Weeks | 1.55 | .85 | 1.576 | 2.101 | .001* | -.16 |  | 1.42 | .77 | 2.044 | 4.215 | .310 | -.05 |  | <.001** | -.09 |
|  | 52 Weeks | 1.41 | .85 | 2.262 | 4.804 |  |  |  | 1.40 | .82 | 2.455 | 6.277 |  |  |  | .936 | .00 |
| Long Exercises Past Half Year | 26 Weeks | 1.32 | .70 | 2.505 | 6.486 | .774 | .01 |  | 1.25 | .70 | 3.184 | 10.574 | .639 | -.01 |  | .001* | -.08 |
|  | 52 Weeks | 1.28 | .77 | 3.145 | 10.016 |  |  |  | 1.25 | .74 | 3.296 | 11.244 |  |  |  | .671 | -.01 |
| Short Exericises Past 4 Weeks | 26 Weeks | 1.33 | .66 | 2.418 | 6.907 | .141 | -.07 |  | 1.31 | .73 | 2.722 | 7.766 | .889 | -.01 |  | .016 | -.05 |
|  | 52 Weeks | 1.29 | .69 | 2.893 | 9.061 |  |  |  | 1.33 | .80 | 2.857 | 8.363 |  |  |  | .872 | .00 |
| Long Exercises Past 4 Weeks | 26 Weeks | 1.22 | .57 | 3.189 | 11.780 | .488 | .03 |  | 1.21 | .67 | 3.636 | 13.834 | .874 | -.01 |  | .009 | -.06 |
|  | 52 Weeks | 1.19 | .61 | 3.749 | 15.159 |  |  |  | 1.24 | .75 | 3.466 | 12.187 |  |  |  | .444 | .02 |

*P-value ≤ .01, **P-value ≤ .001 after Holm-Bonferroni Sequential Correction

^a^Min-Max:1-5 for all variables

**Supplementary Table 3.** *Indirect Effects via Intention*

|  |  | Mindfulness Practice  Short Exercises Weeks 10-26 | | | | | Relaxation Practice  Short Exercises Weeks 10-26 | | | |
| --- | --- | --- | --- | --- | --- | --- | --- | --- | --- | --- |
|  |  | β  [CI] | ***p*** | | *b*  [CI] | ***p*** | β  [CI] | ***p*** | *b*  [CI] | ***p*** |
| Indirect |  |  |  |  |  |  |  |  |  |  |
| Positive Outcome  Expectancies |  | .05  [.03, .07] | .000 | | .05  [.03, .07] | .000 | .06  [.03, .09] | .000 | .05  [.02, .07] | .000 |
| Negative Outcome  Expectancies |  | -.04  [-.07, -.02] | .000 | | -.03  [-.05, -.02] | .000 | -.03  [-.05, -.01] | .015 | -.02  [-.04, -.00] | .017 |
| Injunctive Norms |  | .05  [.03, .08] | .000 | | .04  [.02, .06] | .000 | .03  [.01, .05] | .001 | .02  [.01, .04] | .000 |
| Descriptive Norms |  | .13  [.09, .17] | .000 | | .10  [.07, .14] | .000 | .13  [.09, .18] | .000 | .09  [.06, .12] | .000 |
| Perceived Behavioral Control |  | -.01  [-.02, .01] | .314 | | -.01  [-.03, .01] | .319 | .01  [-.00, .03] | .112 | .01  [-.00, .03] | .112 |
